# Supplementary material for: Evaluating the content validity of two versions of an instrument used in measuring pediatric pain knowledge and attitudes in the Ghanaian context
Source: PLoS One. 2020 Nov 6;15(11):e0241983. doi: 10.1371/journal.pone.0241983 (PMC7647094; doi:10.1371/journal.pone.0241983)
Supplement: S1 Appendix — (DOCX) [file pone.0241983.s001.docx]

**S1 Appendix. Revised Pediatric Nurses’ Knowledge and Attitudes Survey regarding pain (r-PNKAS)**

1. Observable changes in vital signs must be relied upon to verify a child’s/ adolescent’s statement that he/ she has severe pain.

a. True

b. **False**

2. Because of an underdeveloped neurological system, children under 2 years of age have decreased pain sensitivity and limited memory of painful experiences.

a. True

b. **False**

3. If the infant/ child/ adolescent can be distracted from his/ her pain, this usually means that he/ she is not experiencing a high level of pain.

a. True

b. **False**

4. Infants/ children/ adolescents may sleep in spite of severe pain.

a. **True**

b. False

5. Comparable stimuli in different people produce the same intensity of pain.

a. True

b. **False**

6. Ibuprofen and other nonsteroidal anti-inflammatory agents are NOT effective analgesics for bone pain caused by metastases.

a. True

b. **False**

7. *Evidence-based non-drug interventions are very effective for mild-moderate pain control but are rarely helpful for more severe pain.*

*a. True*

*b.* ***False***

8. Children who will require repeated painful procedures (e.g., daily blood draws) should receive maximum treatment for the pain and anxiety of the first procedure to minimize the development of anticipatory anxiety before subsequent procedures.

a. **True**

b. False

9. Respiratory depression rarely occurs in children/ adolescents who have been receiving opioids over a period of months.

a. **True**

b. False

10. Acetaminophen 650 mg PO is approximately equal in analgesic effect to codeine 32 mg PO.

a. **True**

b. False

11. *Combining analgesics (e.g. using acetaminophen, topical anesthetics) and non-drug therapies (e.g. sucrose, and non-nutritive sucking) that work by different mechanisms may result in better pain control with fewer side effects than using a single analgesic agent.*

*a.* ***True***

*b. False*

12. The usual duration of analgesia of morphine IV is 4-5 hours.

a. True

b. **False**

13. Research shows that promethazine (Phenergan®) is a reliable potentiator of opioid analgesics.

a. True

b. **False**

14. Parents should not be present during painful procedures.

a. True

b. **False**

15. Adolescents with a history of substance abuse should not be given opioids for pain because they are at high risk for repeated addiction.

a. True

b. **False**

16. Beyond a certain dosage of morphine, increases in dosage will NOT provide increased pain relief.

a. True

b. **False**

17. Young infants, less than 6 months of age, cannot tolerate opioids for pain relief.

a. True

b. **False**

18. The child/ adolescent with pain should be encouraged to endure as much pain as possible before resorting to a pain relief measure.

a. True

b. **False**

19. *Most children as young as 4 years of age can reliably report pain intensity using a developmentally appropriate self-report tool.*

*a.* ***True***

*b. False*

20. Based on one’s religious beliefs, a child/ adolescent may think that pain and suffering is necessary.

a. **True**

b. False

21. Anxiolytics, sedatives and barbiturates are appropriate medications for the relief of pain during painful procedures.

a. True

b. **False**

22. After the initial recommended dose of opioid analgesic, subsequent doses should be adjusted in accordance with the individual patient’s response.

a. **True**

b. False

23. The child/ adolescent should be advised to use non-drug techniques alone rather than concurrently with pain medications.

a. True

b. **False**

24. Giving children/ adolescents sterile water by injection (placebo) is often a useful test to determine if the pain is real.

a. True

b. **False**

25. In order to be effective, heat and cold should be applied directly to the painful area.

a. True

b. **False**

26. The recommended route of administration of opioid analgesics to children with prolonged cancer-related pain is:

a. Intravenous

b. Intramuscular

c. Subcutaneous

d. **Oral**

e. Rectal

f. I don’t know

27. The recommended route of administration of opioid analgesics to children with brief, severe pain of sudden onset (e.g., trauma or postoperative pain) is:

a. **Intravenous**

b. Intramuscular

c. Subcutaneous

d. Oral

e. Rectal

f. I don’t know

28. Which of the following analgesic medications is considered the drug of choice for the treatment of prolonged moderate to severe pain for children with cancer?

a. Brompton’s cocktail

b. Codeine

c. **Morphine**

d. Meperidine (Demerol)

e. Methadone

f. I don’t know

29. Which of the following IV doses of morphine administered would be equivalent to 15 mg of oral morphine?

a. Morphine 3 mg IV

b. **Morphine 5 mg IV**

c. Morphine 10 mg IV

d. Morphine 15 mg IV

30. Analgesics for post-operative pain should initially be given:

a. **Around the clock on a fixed schedule**

b. Only when the child/ adolescent asks for the medication

c. Only when the nurse determines that the child/ adolescent has moderate or greater discomfort

31. A child with chronic cancer pain has been receiving daily opioid analgesics for 2 months. The doses increased during this time period. Yesterday, the child was receiving morphine 20 mg/ hour intravenously. Today, he has been receiving 25 mg/hour intravenously for 3 hours. The likelihood of the child developing clinically significant respiratory depression is:

a. **< 1%**

b. 1-10%

c. 11-20%

d. 21-40%

e. > 41%

32.Analgesia for chronic cancer pain should be given:

a. **Around the clock on a fixed schedule**

b. Only when the child/ adolescent asks for the medication

c. Only when the nurse determines that the child has moderate or greater discomfort

33.The most likely explanation for why a child/ adolescent with pain would request increased doses of pain medication is:

a. **The child/ adolescent is experiencing increased pain**

b. The child/ adolescent is experiencing increased anxiety or depression

c. The child/ adolescent is requesting more staff attention

d. The child’s/ adolescent’s requests are related to addiction

34. Which of the following drugs are useful for treatment of cancer pain?

a. Ibuprofen (Motrin)

b. Hydromorphone (Dilaudid)

c. Amitriptyline (Elavil)

d. **All of the above**

35.The most accurate judge of the intensity of the child’s/ adolescent’s pain is:

a. The treating physician

b. The child’s/ adolescent’s primary nurse

c. **The child/ adolescent**

d. The pharmacist

e. The child’s/ adolescent’s parent

36.*Which of the following describes the best approach for cultural considerations in caring for a child/adolescent in pain?*

*a. There are no longer cultural influences on the pain experience due to the diversity of the population*

*b. Nurses/ healthcare providers should use knowledge that has defined clearly the influence of pain on culture*

*c.* ***Children/ adolescents should be individually assessed to determine cultural influences on pain***

37.*Children generally over report their pain.*

*a. True*

*b.* ***False***

38.Narcotic/ opioid addiction is defined as psychological dependence accompanied by overwhelming concern with obtaining and using narcotics for psychic effect, not for medical reasons. It may occur with or without the physiological changes of tolerance to analgesia and physical dependence (withdrawal). Using this definition, how likely is it that opioid addiction will occur as a result of treating pain with opioid analgesics? Circle the number closest to what you consider the correct answer.

a. **<1%**

b. 5%

c. 25%

d. 50%

e. 75%

f. 100%

39.On the patient’s record you must mark his pain on the scale below. Circle the number that represents your assessment of Andrew’s pain.

0 1 2 3 4 5 6 7 **8** 9 10

No pain/ discomfort Worst pain/ discomfort

40.Your assessment, above, is made two hours after he received morphine 2 mg IV. After he received the morphine, his pain ratings every half-hour ranged from 6 to 8 and he had no clinically significant respiratory depression, sedation, or other untoward side effects. He has identified 2 as an acceptable level of pain relief. His physician’s order for analgesia is “morphine IV 1-3 mg 1h PRN pain relief.” Check the action you will take at this time.

a. Administer no morphine at this time

b. Administer morphine 1 mg IV now

c. Administer morphine 2 mg IV now

d. **Administer morphine 3 mg IV now**

41.On the patient’s record you must mark his pain on the scale below. Circle the number that represents your assessment of Robert’s pain:

0 1 2 3 4 5 6 7 **8** 9 10

No pain/ discomfort Worst pain/discomfort

42.Your assessment, above, is made two hours after he received morphine 2 mg IV. After he received the morphine, his pain ratings every half-hour ranged from 6 to 8 and he had no clinically significant respiratory depression, sedation, or other untoward side effects. He has identified 2 as an acceptable level of pain relief. His physician’s order for analgesia is “morphine IV 1-3 mg 1h PRN pain relief.” Check the action you will take at this time:

a. Administer no morphine at this time

b. Administer morphine 1 mg IV now

c. Administer morphine 2 mg IV now

d. **Administer morphine 3 mg IV now**

**Note:** The correct answers to the question items have been boldened. Modified items have been italicized for easy identification.
