# Supplementary material for: Evaluating the content validity of two versions of an instrument used in measuring pediatric pain knowledge and attitudes in the Ghanaian context
Source: PLoS One. 2020 Nov 6;15(11):e0241983. doi: 10.1371/journal.pone.0241983 (PMC7647094; doi:10.1371/journal.pone.0241983)
Supplement: S2 Appendix — (DOCX) [file pone.0241983.s002.docx]

**S2 Appendix. Revised Pediatric Healthcare Providers’ Knowledge and Attitudes Survey Regarding Pain (r-PHPKASRP)**

Q1_Observable changes in vital signs must be relied upon to verify a child’s/ adolescent’s self-report of severe pain.

a. True

b. **False**

Q2_Because their nervous system is underdeveloped, children under 2 years of age have decreased pain sensitivity and limited memory of painful experiences.

a. True

b. **False**

Q3_Pediatric patients (infants, children, adolescents) who can be distracted from pain usually do not have severe pain.

a. True

b. **False**

Q4_The usual duration of analgesia of morphine IV is 4-5 hours.

a. True

b. **False**

Q5_Comparable stimuli in different people produce the same intensity of pain.

a. True

b. **False**

Q6_Children who will require repeated painful procedures (e.g., daily blood draws), should receive maximum treatment for the pain and anxiety of the first procedure to minimize the development of anticipatory anxiety before subsequent procedures.

a. **True**

b. False

Q7_Respiratory depression rarely occurs in children/ adolescents who have been receiving stable doses of opioids over a period of months.

a. **True**

b. False

Q8_Infants/ children/ adolescents may sleep in spite of severe pain.

a. **True**

b. False

Q9_Ibuprofen and other nonsteroidal anti-inflammatory agents are NOT effective analgesics for pain from bone metastases.

a. True

b. **False**

Q10_*Evidence-based non-drug interventions are very effective for mild-moderate pain control but are rarely helpful for more severe pain.*

*a. True*

*b.* ***False***

Q11_*Combining analgesics (e.g. using acetaminophen, topical anesthetics) and non-drug therapies (e.g. sucrose, non-nutritive sucking) that work by different mechanisms may result in better pain control with fewer side effects than using a single analgesic agent.*

*a.* ***True***

*b. False*

Q12_Benzodiazepines do not reliably potentiate the analgesia of opioids unless the pain is related to muscle spasms.

a. True

b. **False**

Q13_Parents should not be present during painful procedures.

a. True

b. **False**

Q14_Adolescents with a history of substance abuse should not be given opioids for pain because they are at high risk for repeated addiction.

a. True

b. **False**

Q15_Beyond a certain dosage of morphine, increases in dosage will NOT provide increased pain relief.

a. True

b. **False**

Q16_Young infants, less than 6 months of age, cannot tolerate opioids for pain relief.

a. True

b. **False**

Q17_Spiritual beliefs may lead a child /adolescent to think that pain and suffering are necessary.

a. **True**

b. False

Q18_The child/ adolescent with pain should be encouraged to endure as much pain as possible before resorting to an opioid for pain relief.

a. True

b. **False**

Q19_*Most children as young as 4 years of age can reliably report pain intensity using a developmentally appropriate self-report tool.*

*a.* ***True***

*b. False*

Q20_Anxiolytics, sedatives and barbiturates are appropriate medications for the relief of pain during painful procedures.

a. True

b. **False**

Q21_After the initial dose of opioid analgesic is given, subsequent doses should be adjusted based on the individual patient’s response.

a. **True**

b. False

Q22_The child/ adolescent should be advised to use non-drug techniques alone rather than concurrently with pain medications.

a. True

b. **False**

Q23_Giving children/ adolescents sterile water by injection (placebo) is often a useful test to determine if the pain is real.

a. True

b. **False**

Q24_Sedation always precedes opioid related respiratory depression.

a. **True**

b. False

Q25_Opioid/ narcotic addiction is defined as a chronic neurobiological disease, characterized by impaired control over drug use, compulsive use, continued use despite harm, and craving. It may occur with or without the physiological changes of tolerance to analgesia and physical dependence (withdrawal). Given this information, all children /adolescents whose pain have been treated with opioids for longer than a month are addicted to opioids.

a. True

b. **False**

Q26_The recommended route of administration of opioid analgesics to children with prolonged cancer-related pain is:

a. Intravenous

b. Intramuscular

c. Subcutaneous

d. **Oral**

e. Rectal

f. I don't know

Q27_The usual time to peak effects for traditional analgesics (acetaminophen, non-steroidal anti-inflammatory drugs, and opioids given orally is:

a. 15 minutes

b. 30 minutes

c. **60 minutes**

d. 4 hours

Q28_ The recommended route administration of opioid analgesics to children with brief, severe pain of sudden onset, e.g., trauma or postoperative pain, is:

a. **Intravenous**

b. Intramuscular

c. Subcutaneous

d. Oral

e. Rectal

f. I don't know

Q29_ Which of the following analgesic medications is considered the drug of choice for the treatment of prolonged moderate to severe pain for children with cancer?

a. Acetaminophen

b. Codeine

c. **Morphine**

d. Meperidine (Demerol)

e. I don't know

Q30_ Which of the following IV morphine doses is approximately equivalent to 15 mg of oral morphine?

a. Morphine 3 mg IV

b. **Morphine 5 mg IV**

c. Morphine 10 mg IV

d. Morphine 15 mg IV

Q31_Analgesics for post-operative pain should initially be given:

a. **Around the clock on a fixed schedule**

b. Only when the child/ adolescent asks for the medication

c. Only when the nurse determines that the child/ adolescent has moderate or greater discomfort

Q32_ Analgesia for chronic cancer pain should be given:

a. **Around the clock on a fixed schedule**

b. Only when the child/ adolescent asks for the medication

c. Only when the nurse determines that the child has moderate or greater discomfort

Q33_The most likely reason a child/ adolescent with pain would request increased doses of pain medication is:

a. **The child/ adolescent is experiencing increased pain**

b. The child/ adolescent is experiencing increased anxiety or depression

c. The child/ adolescent is requesting more staff attention

d. The child’s/ adolescent’s requests are related to addiction

Q34_Which of the following drugs are potentially useful for treatment of children’s cancer pain?

a. Non-steroidal anti-inflammatory drugs (NSAIDs)

b. Opioid analgesics

c. Anti-depressants

d. Anti-convulsants

e. **All of the above**

Q35_The most accurate judge of the intensity of the child’s/ adolescent’s pain is the:

a. Treating physician

b. Child's/ adolescent's primary nurse

c. **Child/ adolescent**

d. Pharmacist

e. Child's/ adolescent's parent

Q36_*Which of the following describes the best approach for cultural considerations in caring for a child/adolescent in pain?*

*a. There are no longer cultural influences on the pain experience due to the diversity of the population*

*b. Healthcare providers should use knowledge that has defined clearly the influence of pain on culture*

*c.* ***Children/ adolescents should be individually assessed to determine cultural influences on pain***

Q37_*Children generally over report their pain.*

*a. True*

*b.* ***False***

Q38_On the patient’s record you must mark his pain on the scale below. Choose the number that represents your assessment of Andrew’s pain. *(8)*

0 1 2 3 4 5 6 7 **8** 9 10

No pain/ discomfort Worst pain/discomfort

Q39_Your assessment, above, is made two hours after he received morphine 2 mg IV. After he received the morphine, his pain ratings every half-hour ranged from 6 to 8 and he had no clinically significant respiratory depression, sedation, or other untoward side effects. He has identified 2 as an acceptable level of pain relief. His physician’s order for analgesia is “morphine IV 1-3 mg q1h PRN pain relief.” Check the action you will take at this time.

a. Administer no morphine at this time

b. Administer morphine 1 mg IV now

c. Administer morphine 2 mg IV now

d. **Administer morphine 3 mg IV now**

Q40_Select the number that represents your assessment of Robert’s pain: *(8)*

0 1 2 3 4 5 6 7 **8** 9 10

No pain/ discomfort Worst pain/discomfort

Q41_Your assessment, above, is made two hours after he received morphine 2 mg IV. After he received the morphine, his pain ratings every half-hour ranged from 6 to 8 and he had no clinically significant respiratory depression, sedation, or other untoward side effects. He has identified 2 as an acceptable level of pain relief. His order for analgesia is “morphine IV 1-3 mg q1h PRN pain relief.” Check the action you will take at this time:

a. Administer no morphine at this time

b. Administer morphine 1 mg IV now

c. Administer morphine 2 mg IV now

d. **Administer morphine 3 mg IV now**

**Note:** The correct answers to the question items have been boldened. Modified items have been italicized for easy identification.
