# Supplementary material for: Evaluating the content validity of two versions of an instrument used in measuring pediatric pain knowledge and attitudes in the Ghanaian context
Source: PLoS One. 2020 Nov 6;15(11):e0241983. doi: 10.1371/journal.pone.0241983 (PMC7647094; doi:10.1371/journal.pone.0241983)
Supplement: Attachment — Submitted filename: Review manuscript PONE-D-20-05637 VMS.docx [file pone.0241983.s004.docx]

Review manuscript

PONE-D-20-05637, entitled "Comparison of the content validity of two instruments used in measuring pediatric pain knowledge and attitudes" PLOS ONE

**Reviewer:** Vicente Miñana-Signes

# 1 Summary of the research and your overall impression

## 1.1 Reviewer comment:

The manuscript compared the content validity of two instruments used in measuring nurse pediatric pain knowledge and attitudes.

As strong points of the article, it should be noted that they have had a wide participation of experts. Furthermore, it appears that the author of the questionnaire, Manworren, has been involved in the counseling process.

As weaknesses, the authors acknowledge both in the limitations section and in the conclusions that, as future lines, a validation of the criterion and the construct is required.

From my point of view, we must differentiate between (Lobiondo-Wood G, Haber J.; 2013)^[1]^:

- The **content validity**. Content validity evaluates qualitatively whether the questionnaire covers all the dimensions of the phenomenon that wants to measure, since it is considered that an instrument is valid in its content if contemplates all related aspects with the concept that measures. The researcher begins by defining the concept and identifying the attributes or dimensions of the concept. The items that reflect the concept and its domain are developed.
- The **apparent validity**. It is a subtype of content validity. It is a face validity, which is a rudimentary type of validity that basically verifies that the instrument fives the appearance of measuring the concept. It is an intuitive type of validity in which colleagues or subjects are asked to read the instrument and evaluate the content in terms of whether it appear to reflect the concept the researcher intends to measure. Or if the elements included in an instrument are relevant.
- **Criterion validity**. It is the degree of correlation between an instrument and another measure of the variable under study that serves as criterion or reference.
- **Construct validity**. It is understood as the degree to which an instrument measures the bipolar evaluative dimension for which it was designed.

Actually, in the present article, what they do is to measure **apparent validity**. It is important because the acceptance of a scale by several people gives consistency when using it. However, apparent and content validity is a relevant method especially when designing an instrument. It is not so important when the instrument has been previously validated and used in different areas.

On the other hand, and as the authors say, these tools have already been validated and used in various studies. Knowing that its content validity has already been reviewed as they describe and explain. In that case, why have they revised the apparent validity again? Why haven't they gone a step further? Why have the criteria not been applied and reviewed?

For these reasons, I think the article does not reach the level required for a journal like PLOS ONE, and should be rejected.

From my point of view, the approach and content is well developed, but due to the characteristics of the tools used, these require a criterion validity, a construct validity, or a cross-cultural validation.

The authors summarize the main research question and key findings. Even, the authors identify other literature on the topic and explain how the study relates to this previously published research.

However, I would like to make some specific suggestions in the next point.

# 2 Discussion of specific areas for improvement

## 2.1 Major issues

### 2.1.1 Reviewer comment:

Suggestions for improvement do not refer to major issues

## 2.2 Minor issues

### 2.2.1 Reviewer comment:

#### 2.2.1.1 Title

As a suggestion, and to contextualize more the article from the first moment, the title could refer to Pediatric Healthcare Providers' (HCPs) or pediatric nurses as well as keywords.

In fact, HCPs are referenced every time in the page 3, line 61 paragraph.

Another more clarifying explanation is found in page 3 and line 68: “These two instruments were developed to measure healthcare professionals and students’ knowledge and attitudes regarding children’s pain [13].” But it is still a personal appreciation.

#### 2.2.1.2 Abstract and introduction

Page 2; line 24. The introduction does not set the stage adequately. As in the title, it is required to specify who the study is aimed at. To miss this information may decontextualize and imply the study population.

In the abstract, the authors explain why the study matters and put the research in context properly. However:

In page 2; line 25; the authors clarify that “*This was considered necessary due to the universal differences in* ***culture, semantics and healthcare resources*** *in different parts of the globe*”, but in the following paragraph:

In page 2; line 31; they specify that the experts only will check **the relevance** and **clarity** of the items will be reviewed **without mention** culture, semantics and healthcare resources. If it is so important, because you mention it at the beginning of the abstract, could the experts have been asked about the changes due to the cultural factor? Has any change been made due to semantic and cultural changes?

Content validity is a relevant method especially when designing an instrument. It is not so important when the instrument has been previously validated and used in different areas. However, when an instrument is translated into another language, if the explored concepts are supposed to change significantly from one culture to another, it may be useful to recheck the face validity.

Moreover, in page 4, line 92 It is said that validity is not the property of an instrument, but depends on the interpretation related to the context and participants. However, knowledge of health is based on science, evidence, principles, theories, and is universal. Therefore, they do not depend on the interpretation of people and cultures.

Content validity evaluates qualitatively whether the questionnaire covers all the dimensions of the phenomenon to be measured, since an instrument is considered to be valid in its content if it considers all aspects related to the concept it measures. For this, it is necessary to have a clear idea of the conceptual aspects to be measured. And in this case, since the instrument had previously been validated and used in different areas, the content was already available.

Page 3; line 67. This is what I cannot understand. In this section, the questions that arise are: When talking about the revised version (PHPKASRP). Is the article review being done for the first time in this article? Or has this version already been created before? Why do you call short version if they have almost the same number of questions?...May be could you give more details or change the way to explain it.

In the next sentence: page 3; line 68. “These **two instruments** were developed to measure healthcare professionals and students’ knowledge and attitudes regarding children’s pain (**13**)”. We find the reference of the PNKAS, what about the PHPKASRP reference? Could you explain this better?

When talking about instrument validation, I would give more information about whether or not PHPKASRP is validated, etc.

Page 4; line 84. References for PHPKASRP are again needed.

#### 2.2.1.3 Figures and tables

The information in Tables 1 and 3 are explained in the text. Table 1 could be omitted by completing the information in the text. However, Table 3 can be kept, since it serves as a synthesis of the results.

#### 2.2.1.4 Methods

Page 6; paragraph line 128-133.There is no specific reference to cultural and semantic factors. Despite the emphasis that has been given in the introduction by providing references.

They talk about: comprehensiveness, objectivity, organization and relevance defined as “comprehensive: that issue containing important information to reach the objective of the study, stated in a comprehensible manner; Objective: that issue which is easy to understand; organization: the disposition of the issues and alternatives as well as their content; Relevant: that question which is related to achieving the goal of the research”

Page 8; line 182. Again, the article reference of the revised version (PHPKASRP instrument) is missed.

#### 2.2.1.5 Results, discussion, conclusions

The results on page 10 should reference Table 3, where the results are summarized.

The authors acknowledge both the limitations and the conclusions that, as future lines, a validation of the criterion and the construct is required.

#### 2.2.1.6 Statistical analysis

This type of validity requires basic statistics. Experts and / or researchers use in evaluating the relevance of a scale that requires descriptive analysis.

# 3 Other Things to Check

Here are some additional things to look at when you’re reading.

## 3.1 Writing quality & clarity

### 3.1.1 Reviewer comment:

## 3.2 Plagiarism

### 3.2.1 Reviewer comment:

## 3.3 Reference list

### 3.3.1 Reviewer comment:

## 3.4 Wrapping Up

### 3.4.1 Reviewer comment:

## 3.5 Any other points

### 3.5.1 Reviewer comment:

1. Lobiondo-Wood G, Haber J. Nursing research: methods and critical appraisal for evidence-based practice. 8th ed. St. Louis: Mosby; 2013.
