# Supplementary material for: Evaluating the content validity of two versions of an instrument used in measuring pediatric pain knowledge and attitudes in the Ghanaian context
Source: PLoS One. 2020 Nov 6;15(11):e0241983. doi: 10.1371/journal.pone.0241983 (PMC7647094; doi:10.1371/journal.pone.0241983)
Supplement: Attachment — Submitted filename: PLOS ONE.docx [file pone.0241983.s005.docx]

To be accepted for publication in PLOS ONE, research articles must satisfy the following criteria:

1. The study presents the results of original research.

Yes

2. Results reported have not been published elsewhere.

Yes

3. Experiments, statistics, and other analyses are performed to a high technical standard and are described in sufficient detail.

In parts. There was no review of the validity of the content of these instruments and the lack of reliability analysis (internal consistency, test-retest, intra-evaluator). In addition, the analysis of casual agreement among experts, with the Kappa statistical coefficient.

4. Conclusions are presented in an appropriate fashion and are supported by the data.

Sim.

5. The article is presented in an intelligible fashion and is written in standard English.

Sim

6. The research meets all applicable standards for the ethics of experimentation and research integrity.

Sim

7. The article adheres to appropriate reporting guidelines and community standards for data availability.

Sim

Comments:

This descriptive methodological study was carried out with the objective of evaluating and comparing the content validity of two instruments of knowledge and attitude about pediatric pain.

The study was well written and relevant, however, my main concerns are related to Validity analysis. The lack of review of the validity of the content of these instruments and the lack of reliability analysis (internal consistency, test-retest, intra-evaluator). In addition, the analysis of casual agreement among experts, with the Kappa statistical coefficient.

Type of study: Review the written form of the type of study. "Descriptive methodological study with content validity".

Review confusing writing in the first paragraph of the results.
