# Supplementary material for: Evaluating the content validity of two versions of an instrument used in measuring pediatric pain knowledge and attitudes in the Ghanaian context
Source: PLoS One. 2020 Nov 6;15(11):e0241983. doi: 10.1371/journal.pone.0241983 (PMC7647094; doi:10.1371/journal.pone.0241983)
Supplement: Attachment — Submitted filename: PONE-D-20-05637 Review.docx [file pone.0241983.s006.docx]

**REVIEWER COMMENTS ON MANUSCRIPT “Comparison of the content validity of two instruments used in measuring pediatric pain knowledge and attitudes”**

**(PONE-D-20-05637)**

**Dear Editor**

Thank you for the opportunity to review this manuscript. Please find below my comments.

**General comments:**

The manuscript aims to evaluate and compare the content validity of PNKAS and its revised version, PHPKASRP from a Ghanaian perspective. I have read the manuscript with careful attention. First of all, I was expecting to find the revised the PNKAS and PHPKASRP) as appendices. I think it is worthy to include them as appendices since they are the final products of the study. I have other comments for better improvement.

**Title**

The title of the manuscript reads as if the study compares completely different instruments. However, PHPKASRP is a revised version of PNKAS, and it would be not surprising to find high similarity between the two versions in terms of content validity. Therefore, it would be irrational making a comparison between two versions of the same survey. Further, the title does not indicate that the assessment of content validity was performed in Ghanaian context.

I would suggest revising the title to address these two comments. For example “Evaluating the content validity of two instruments used in measuring pediatric pain knowledge and attitudes in Ghanaian context”.

**Keywords**

Since the keyword “psychometric evaluation” might indicates reliability, validity, and/or responsiveness, the authors only examined the content validity. I believe this keyword would be misleading. Please replace it with “content validity”. Furthermore, I would suggest including “Ghana” as a keyword.

**Abstract**

Line 24-25. “*we compared the content validity of two instruments used in measuring pediatric pain knowledge and attitudes*”

Again, this sentence reads as if the authors compared completely different instruments, and it does not indicate that the evaluation was conducted in Ghanaian context. I would suggest recast this sentence as “we evaluated the content validity of two versions of an instrument used in measuring pediatric pain knowledge and attitudes in the Ghanaian context”.

Line 28-30. “*Thirteen (13) pediatric experts in Ghana assessed the content validity of two instruments: the 42-item Pediatric Nurses' Knowledge and Attitudes Survey Regarding Pain (PNKAS) and the 41-item Pediatric Healthcare Providers’ Knowledge and Attitudes Survey Regarding Pain (PHPKASRP).*”

Same here. I suggest recast this sentence as “Thirteen (13) pediatric experts in Ghana assessed the content validity of the 42-item Pediatric Nurses' Knowledge and Attitudes Survey Regarding Pain (PNKAS) and its revised version, the 41-item Pediatric Healthcare Providers’ Knowledge and Attitudes Survey Regarding Pain (PHPKASRP).

If you accept this suggestion, please modify the rest of abstract and manuscript to be consistent with these changes.

Line 31. “*four-point likert scaled*”

Please capitalize the letter “L” in “Likert”.

**Introduction**

Line 66-68. “*Key among them are Pediatric Nurses’ Knowledge and Attitudes Survey regarding pain (PNKAS) and its revised shorter version, the Pediatric Healthcare Providers’ Knowledge and Attitudes Survey Regarding Pain (PHPKASRP)*”

I know the PNKAS was published by Manworren RC (reference 13). However, was the PHPKASRP also published in the same study (reference 13)? Either way, you need to cite the study/studies that published the PNKAS and PHPKASRP in the above sentence.

Further, since the difference between the two versions in terms of number of items is one item only (42 vs 41), I am not sure if it is appropriate to describe the PHPKASRP as “revised shorter version”. I suggest deleting “shorter” form the above sentence.

Line 68-70. “*These two instruments were developed to measure healthcare professionals and students’ knowledge and attitudes regarding children’s pain [13]*”

This sentence reads as if Manworren RC (reference 13) published the two instruments in their study. However, I am not sure if this is true. Please make sure and correct the reference accordingly.

Line 72. “*Following the development of the PNKAS instrument*”

No need to follow the PNKAS or the PHPKASRP with the word “instrument” since the last word both terms is “survey”. Please delete “instrument” from this sentence and throughout the manuscript if you accept.

Line 73-78. “*Face and content validity of the instrument was established by five pain management experts in the United States of America. Internal consistency of the PNKAS instrument has also been assessed by two distinct groups with a reported acceptable Cronbach’s alpha value of 0.72 among 247 pediatric nurses and 0.77 among 88 members of a children’s nursing organization. Test-retest reliability analysis among 12 clinicians (6 nurses and 6 child life specialists) recorded a correlation coefficient of 0.67, signifying an acceptable level of instrument stability*”.

Too much information are mentioned in this section. You may simply report the psychometric properties without going into further details about the number of participants or experts and their genders.

Line 85. “*(PHPKASRP instrument)”*

Same here. Please see my comment above about the word “instrument”.

Line 85-87. “*Content validity of the PHPKASRP has been established by national content experts comprising of physicians, pediatric nurses and pharmacists in the United States of America*”

Please cite the reference for this sentence.

Line 87-89. “*According to the instrument developer (Manworren, R.C.B.), the instrument has been translated into other languages and permitted for use in many organizations around the world.*”

Same here. Please cite the reference for this sentence.

Line 96-98. “*As part of plans to use a pediatric pain knowledge and attitude instrument as a tool in assessing the effectiveness of a short-course educational program for nursing students and nurses*”

According to this sentence, the content validity of the two versions was examined for the purpose of this course. Will be the course conducted in Ghana? Are all of the audience from Ghana? Or others from different countries will be included?

Line 98. “*the current study sought to evaluate and compare the content validity*”

Again, comparing the content validity of two versions of the same instrument would be irrational. I suggest narrowing your aim to “evaluating” without “comparing”.

**Materials and Methods**

Line 103. “*This descriptive methodological study*”

According to Portney and Watkins (Foundations of Clinical Research: Applications to Practice. Vol. 2. Prentice Hall Upper Saddle River, NJ; 2000), validity studies are described as exploratory methodological studies. Please correct the design.

Line 110-111. “*The experts were chosen based on their level of training, clinical and/ or teaching experience in pediatrics*”.

The criteria of selecting the experts mentioned here is wide and lacks of accuracy. What do you mean by “their level”? Did you have minimum level of experience? Please be more specific.

I think it is very important to be more careful in describing how the experts were selected because they are the main element of this study.

Line 135. “*Both individual item-level and scale level content validity indices*”

There are several issues in the manuscript regarding the use of abbreviations as will be stated in some of the following comments. As a rule of thumb, you should explain each of your abbreviation the first time it appears in the main text, and then use that abbreviation instead of the complete term in the rest of the manuscript.

In this sentence, you mentioned the individual item-level content validity (I-CVI) and the average scale level content validity (S-CVI/Ave) for the first time. Therefore, you should have introduced their abbreviations and use them instead of the complete term in the rest of your manuscript.

Line 149. “*Chicago, IL*”

The correct citation of the SPSS version 25 is (Armonk, NY). Please refer to this webpage for more information (<https://www.ibm.com/support/pages/how-cite-ibm-spss-statistics-or-earlier-versions-spss>).

Line 182-184. “*The Pediatric Nurses' Knowledge and Attitudes Survey Regarding Pain (PNKAS) is a modification of Nurses' Knowledge and Attitudes Survey Regarding Pain (NKASRP)*”.

You do not need to use the complete terms since you have already introduced their abbreviations in the introduction (line 67-68).

Line 191-193. “*Ethical approval for the study with reference number CHRPE/AP/574/18 was provided by the Committee on Human Research, Publications and Ethics (CHRPE), School of Medical Sciences (SMS), Kwame Nkrumah University of Science and Technology (KNUST)*”.

One the other hand, you do not need to state the abbreviations of the Committee on Human Research, Publications and Ethics, School of Medical Sciences, and Kwame Nkrumah University of Science and Technology since they were only mentioned once in the manuscript.

**Results**

Line 205. “*Majority of them were female (69%)*”.

Please correct the percentage to (69.2%) to be consistence with Table 1.

Table 1

Is there is any reason why you used the median instead of the mean to report the central tendency?

Line 215-216. “*The average relevance CVI for the scale (S-CVI/Ave) was 0.87*”.

Same to my previous comments about the abbreviations. Use the abbreviation without introducing it again. Please correct accordingly.

Line 232. “*individual-item content validity index (I-CVI)*”.

Same here regarding the use of complete term.

Table 2.

Some issues on this table need to be addressed:

The table is lengthy and I think it would be better to include it as a supplemental table instead of containing it in the manuscript.

There are four cells that are missing in the last column (Action Taken; Revised Form) of the table.

You mentioned five items were removed from each of the PNKAS and PHPKASRP. It is not clear from the table which items are they. Please specify them.

It is not clear why some items were retained or not revised although their scores were less than 70 and some of them received comments form the experts. For example, the question about respiratory depression and the use of opioids, Questions 18, the question about Anxiolytics, sedatives and barbiturates, Question 24 of the PHPKASRP, and Question 38 of the PNKAS (Q25 on the PHPKASRP). I think the readers deserve to know the reasons behind ignoring the low scores and experts’ comments in making the decision of retaining or not revising those items. Otherwise, the ratings and comments of the experts are meaningless.

Line 254-256. “*In consultation with the instrument developer (Manworren, R.C.B.), the researchers addressed all comments from the experts through a review process*”

This a bit confusing. In the methods section, line 139-142, the authors stated, “items which were < 0.70 were eliminated, revised or kept based on feedback and its importance to the construct under investigation. Items which had an I-CVI value of ≥ 0.70 were kept or modified based on theoretical relevance and/ or the feedback received from the participating pediatric experts”. Nothing were mentioned in the methods about involving the developer in the decision-making process.

Table 3.

What do you mean by “universal agreement”? How this outcome was calculated?

**Discussion**

Line 311-314. “*In line with the recommendations of Delgado-Rico and colleagues [43], decisions on items (i.e., elimination, modification or conservation) were made on the basis of the content validity indices, feedback given by experts, inputs from the instrument developer and the contribution of the items to the overall construct under investigation*”.

Here you mentioned the involvement of the developer in the decision-making process. Again, nothing was mentioned about the developer involvement in the methods. Further, I think you need to cite Delgado-Rico and colleagues as well in your methods since you followed their recommendations in your decisions making.

Line 318-319. “*we recommend the use of the both instruments (PNKAS and PHPKASRP) as simple, easy to use*”

There is no evidence of simplicity nor easiness of use that can be drawn from you results.

**Conclusions**

Line 355. “*Both instruments are simple, easy to use*”

Same here, you need to show the evidence or delete this sentence.

**References**

I do not believe it is required to include the publishers in the reference (e.g., Lippincott, Williams & Wilkins, Elsevier, Hindawi ..etc.). The authors need to format their references according to the journal’s instructions (<https://journals.plos.org/plosone/s/submission-guidelines#loc-references>).

**Other comments**

There are several places throughout the manuscript with extra spaces between the words (e.g., line 39, line 41). Please correct the text.
