# Supplementary material for: Evaluating the content validity of two versions of an instrument used in measuring pediatric pain knowledge and attitudes in the Ghanaian context
Source: PLoS One. 2020 Nov 6;15(11):e0241983. doi: 10.1371/journal.pone.0241983 (PMC7647094; doi:10.1371/journal.pone.0241983)
Supplement: Attachment — Submitted filename: Response to Reviewers.docx [file pone.0241983.s007.docx]

**Response to Review Comments**

Thank you for taking time off your busy schedules and providing us constructive feedback to further improve our manuscript. Please find below our responses to the review comments.

| **Comment** | **Response** |
| --- | --- |
| **Overall Comments from Reviewer 1** |  |
| This descriptive methodological study was carried out with the objective of evaluating and comparing the content validity of two instruments of knowledge and attitude about pediatric pain.  The study was well written and relevant, however, my main concerns are related to Validity analysis. The lack of review of the validity of the content of these instruments and the lack of reliability analysis (internal consistency, test-retest, intra-evaluator). In addition, the analysis of casual agreement among experts, with the Kappa statistical coefficient. | Thank you for the feedback. The review of validity and reliability of the instruments have already been provided on page 4, lines 75-87. |
| **Specific Comments from Reviewer 1** |  |
| Type of study: Review the written form of the type of study. "Descriptive methodological study with content validity". | The study type has been amended on page 5, line 103. |
| Review confusing writing in the first paragraph of the results. | Although it was unclear which sections of the first paragraph of the results you were referring to, changes have been made at this section as found on page 9, lines 199-203. |
| **Overall Comments from Reviewer 2** |  |
| The manuscript aims to evaluate and compare the content validity of PNKAS and its revised version, PHPKASRP from a Ghanaian perspective. I have read the manuscript with careful attention. First of all, I was expecting to find the revised the PNKAS and PHPKASRP) as appendices. I think it is worthy to include them as appendices since they are the final products of the study. I have other comments for better improvement. | Thank you for the feedback. The revised instruments have been provided as appendices (refer to S1 Appendix and S2 Appendix). |
| **Specific Comments from Reviewer 2** |  |
| **Title** |  |
| The title of the manuscript reads as if the study compares completely different instruments. However, PHPKASRP is a revised version of PNKAS, and it would be not surprising to find high similarity between the two versions in terms of content validity. Therefore, it would be irrational making a comparison between two versions of the same survey. Further, the title does not indicate that the assessment of content validity was performed in Ghanaian context.  I would suggest revising the title to address these two comments. For example “Evaluating the content validity of two instruments used in measuring pediatric pain knowledge and attitudes in Ghanaian context”. | As suggested, changes have been made to the titles (long and short) as found on page 1, lines 1-7. |
| **Keywords** |  |
| Since the keyword “psychometric evaluation” might indicates reliability, validity, and/or responsiveness, the authors only examined the content validity. I believe this keyword would be misleading. Please replace it with “content validity”. Furthermore, I would suggest including “Ghana” as a keyword. | Changes have been made on page 3, line 54. |
| **Abstract** |  |
| Line 24-25. “*we compared the content validity of two instruments used in measuring pediatric pain knowledge and attitudes*”  Again, this sentence reads as if the authors compared completely different instruments, and it does not indicate that the evaluation was conducted in Ghanaian context. I would suggest recast this sentence as “we evaluated the content validity of two versions of an instrument used in measuring pediatric pain knowledge and attitudes in the Ghanaian context”. | Changes have been made at the abstract and sections of the manuscript. Refer to page 2, lines 24-25, 28-29 and page 5, line 106. |
| Line 28-30. “*Thirteen (13) pediatric experts in Ghana assessed the content validity of two instruments: the 42-item Pediatric Nurses' Knowledge and Attitudes Survey Regarding Pain (PNKAS) and the 41-item Pediatric Healthcare Providers’ Knowledge and Attitudes Survey Regarding Pain (PHPKASRP).*”  Same here. I suggest recast this sentence as “Thirteen (13) pediatric experts in Ghana assessed the content validity of the 42-item Pediatric Nurses' Knowledge and Attitudes Survey Regarding Pain (PNKAS) and its revised version, the 41-item Pediatric Healthcare Providers’ Knowledge and Attitudes Survey Regarding Pain (PHPKASRP).  If you accept this suggestion, please modify the rest of abstract and manuscript to be consistent with these changes. | Changes have been made at the abstract and sections of the manuscript. Refer to page 2, lines 24-25, 28-29 and page 5, line 106. |
| Line 31. “*four-point likert scaled*”  Please capitalize the letter “L” in “Likert”. | As suggested, this has been changed on page 2, line 32. |
| **Introduction** |  |
| Line 66-68. “*Key among them are Pediatric Nurses’ Knowledge and Attitudes Survey regarding pain (PNKAS) and its revised shorter version, the Pediatric Healthcare Providers’ Knowledge and Attitudes Survey Regarding Pain (PHPKASRP)*”  I know the PNKAS was published by Manworren RC (reference 13). However, was the PHPKASRP also published in the same study (reference 13)? Either way, you need to cite the study/studies that published the PNKAS and PHPKASRP in the above sentence.  Further, since the difference between the two versions in terms of number of items is one item only (42 vs 41), I am not sure if it is appropriate to describe the PHPKASRP as “revised shorter version”. I suggest deleting “shorter” form the above sentence. | Citations have been respectively placed by each instrument, refer to page 4, lines 71-73. Information on the PHPKASRP was provided by the instrument developer (Manworren RCB) through email communication. According to the instrument developer, she is yet to publish information about PHPKASRP.  Also, the word “shorter” has been removed from the sentence, refer to page 4, line 71. |
| Line 68-70. “*These two instruments were developed to measure healthcare professionals and students’ knowledge and attitudes regarding children’s pain [13]*”  This sentence reads as if Manworren RC (reference 13) published the two instruments in their study. However, I am not sure if this is true. Please make sure and correct the reference accordingly. | Modifications have been made on page 3, line 70 and page 4, lines 71-74. |
| Line 72. “*Following the development of the PNKAS instrument*”  No need to follow the PNKAS or the PHPKASRP with the word “instrument” since the last word both terms is “survey”. Please delete “instrument” from this sentence and throughout the manuscript if you accept. | All instances of the word “instrument(s)” which followed the PNKAS and or PHPKASRP have been removed from the manuscript. |
| Line 73-78. “*Face and content validity of the instrument was established by five pain management experts in the United States of America. Internal consistency of the PNKAS instrument has also been assessed by two distinct groups with a reported acceptable Cronbach’s alpha value of 0.72 among 247 pediatric nurses and 0.77 among 88 members of a children’s nursing organization. Test-retest reliability analysis among 12 clinicians (6 nurses and 6 child life specialists) recorded a correlation coefficient of 0.67, signifying an acceptable level of instrument stability*”.  Too much information are mentioned in this section. You may simply report the psychometric properties without going into further details about the number of participants or experts and their genders. | As recommended, this statement has been summarised concisely; refer to page 4, lines 75-79 . |
| Line 85. “*(PHPKASRP instrument)”*  Same here. Please see my comment above about the word “instrument”. | As earlier indicated, all instances of the word “instrument(s)” which followed the PNKAS and or PHPKASRP have been removed from the manuscript. |
| Line 85-87. “*Content validity of the PHPKASRP has been established by national content experts comprising of physicians, pediatric nurses and pharmacists in the United States of America*”  Please cite the reference for this sentence. | Citation to this statement has been provided on page 4, line 87. |
| Line 87-89. “*According to the instrument developer (Manworren, R.C.B.), the instrument has been translated into other languages and permitted for use in many organizations around the world.*”  Same here. Please cite the reference for this sentence. | Citation to this statement has been provided on page 4, line 89. |
| Line 96-98. “*As part of plans to use a pediatric pain knowledge and attitude instrument as a tool in assessing the effectiveness of a short-course educational program for nursing students and nurses*”  According to this sentence, the content validity of the two versions was examined for the purpose of this course. Will be the course conducted in Ghana? Are all of the audience from Ghana? Or others from different countries will be included? | Clarifications on this have been provided at page 5, line 98. |
| Line 98. “*the current study sought to evaluate and compare the content validity*”  Again, comparing the content validity of two versions of the same instrument would be irrational. I suggest narrowing your aim to “evaluating” without “comparing”. | Suggested amendment has been made on page 5, line 98. |
| **Materials and Methods** |  |
| Line 103. “*This descriptive methodological study*”  According to Portney and Watkins (Foundations of Clinical Research: Applications to Practice. Vol. 2. Prentice Hall Upper Saddle River, NJ; 2000), validity studies are described as exploratory methodological studies. Please correct the design. | The study type has been amended on page 5, line 103. |
| Line 110-111. “*The experts were chosen based on their level of training, clinical and/ or teaching experience in pediatrics*”.  The criteria of selecting the experts mentioned here is wide and lacks of accuracy. What do you mean by “their level”? Did you have minimum level of experience? Please be more specific.  I think it is very important to be more careful in describing how the experts were selected because they are the main element of this study. | Specifications about the expertise have been provided on page 5, lines 111-112. |
| Line 135. “*Both individual item-level and scale level content validity indices*”  There are several issues in the manuscript regarding the use of abbreviations as will be stated in some of the following comments. As a rule of thumb, you should explain each of your abbreviation the first time it appears in the main text, and then use that abbreviation instead of the complete term in the rest of the manuscript.  In this sentence, you mentioned the individual item-level content validity (I-CVI) and the average scale level content validity (S-CVI/Ave) for the first time. Therefore, you should have introduced their abbreviations and use them instead of the complete term in the rest of your manuscript.  Line 149. “*Chicago, IL*”  The correct citation of the SPSS version 25 is (Armonk, NY). Please refer to this webpage for more information (<https://www.ibm.com/support/pages/how-cite-ibm-spss-statistics-or-earlier-versions-spss>). | As suggested, amendments have been made throughout the manuscript; refer to page 6, line 134 for an example.  The appropriate citation for SPSS version 25 have been provided on page 7, line 147. |
| Line 182-184. “*The Pediatric Nurses' Knowledge and Attitudes Survey Regarding Pain (PNKAS) is a modification of Nurses' Knowledge and Attitudes Survey Regarding Pain (NKASRP)*”.  You do not need to use the complete terms since you have already introduced their abbreviations in the introduction (line 67-68). | Suggested amendment has been made at page 7, line 156. |
| Line 191-193. “*Ethical approval for the study with reference number CHRPE/AP/574/18 was provided by the Committee on Human Research, Publications and Ethics (CHRPE), School of Medical Sciences (SMS), Kwame Nkrumah University of Science and Technology (KNUST)*”.  One the other hand, you do not need to state the abbreviations of the Committee on Human Research, Publications and Ethics, School of Medical Sciences, and Kwame Nkrumah University of Science and Technology since they were only mentioned once in the manuscript. | As suggested, these abbreviations have been removed; refer to page 8, lines 189-190. |
| **Results** |  |
| Line 205. “*Majority of them were female (69%)*”.  Please correct the percentage to (69.2%) to be consistence with Table 1. | Suggested modification has been provided on page 9, line 201. |
| Table 1  Is there is any reason why you used the median instead of the mean to report the central tendency? | The rationale for the use of median has been provided on page 7, lines 147-149. |
| Line 215-216. “*The average relevance CVI for the scale (S-CVI/Ave) was 0.87*”.  Same to my previous comments about the abbreviations. Use the abbreviation without introducing it again. Please correct accordingly. | As suggested, changes have been made throughout the manuscript. An instance of this can be seen on page 9, lines 209, 214 among others. |
| Line 232. “*individual-item content validity index (I-CVI)*”.  Same here regarding the use of complete term. | Modifications have been provided throughout the manuscript. Instances of this can be seen on page 9, line 219 and page 10, line 223. |
| Table 2.  Some issues on this table need to be addressed:  The table is lengthy and I think it would be better to include it as a supplemental table instead of containing it in the manuscript.  There are four cells that are missing in the last column (Action Taken; Revised Form) of the table.  You mentioned five items **were removed from each of the** PNKAS and PHPKASRP. It is not clear from the table which items are they. Please specify them.  It is not clear why some items were retained or not revised although their scores were less than 70 and some of them received comments from the experts. For example, the question about respiratory depression and the use of opioids, Questions 18, the question about Anxiolytics, sedatives and barbiturates, Question 24 of the PHPKASRP, and Question 38 of the PNKAS (Q25 on the PHPKASRP). I think the readers deserve to know the reasons behind ignoring the low scores and experts’ comments in making the decision of retaining or not revising those items. Otherwise, the ratings and comments of the experts are meaningless. | As suggested, Table 2 has been designated as S1 Table in the supplementary document file named “S1_Table.docx”.  Missing information has been provided at the initial four empty cells of the now “S1 Table”.  Five items were modified from each of the instruments and “not removed”; these items have been specified on page 11, lines 249-251.  Factors which influenced the decisions made on items have been provided on page 6, lines 138-140. |
| Line 254-256. “*In consultation with the instrument developer (Manworren, R.C.B.), the researchers addressed all comments from the experts through a review process*”  This a bit confusing. In the methods section, line 139-142, the authors stated, “items which were < 0.70 were eliminated, revised or kept based on feedback and its importance to the construct under investigation. Items which had an I-CVI value of ≥ 0.70 were kept or modified based on theoretical relevance and/ or the feedback received from the participating pediatric experts”. Nothing were mentioned in the methods about involving the developer in the decision-making process. | This has been rectified on page 6, lines 138-140. |
| Table 3.  What do you mean by “universal agreement”? How this outcome was calculated? | Definition and calculation of “universal agreement” has been provided on page 6, lines 141-142. |
| **Discussion** |  |
| Line 311-314. “*In line with the recommendations of Delgado-Rico and colleagues [43], decisions on items (i.e., elimination, modification or conservation) were made on the basis of the content validity indices, feedback given by experts, inputs from the instrument developer and the contribution of the items to the overall construct under investigation*”.  Here you mentioned the involvement of the developer in the decision-making process. Again, nothing was mentioned about the developer involvement in the methods. Further, I think you need to cite Delgado-Rico and colleagues as well in your methods since you followed their recommendations in your decisions making. | As earlier indicated, these concerns have been addressed, refer to page 6, lines 138-140. |
| Line 318-319. “*we recommend the use of the both instruments (PNKAS and PHPKASRP) as simple, easy to use*”  There is no evidence of simplicity nor easiness of use that can be drawn from you results. | These descriptions have been removed; refer to page 12, lines 288-289. |
| **Conclusions** |  |
| Line 355. “*Both instruments are simple, easy to use*”  Same here, you need to show the evidence or delete this sentence. | Suggested modifications have been made on page 13, lines 304-305. |
| **References** |  |
| I do not believe it is required to include the publishers in the reference (e.g., Lippincott, Williams & Wilkins, Elsevier, Hindawi ..etc.). The authors need to format their references according to the journal’s instructions (<https://journals.plos.org/plosone/s/submission-guidelines#loc-references>). | All instances of the “publishers” have been removed to comply with the journal requirements; refer to pages 14-18. |
| **Other comments** |  |
| There are several places throughout the manuscript with extra spaces between the words (e.g., line 39, line 41). Please correct the text. | Extra spaces have been removed from the entire manuscript. |
| **Overall Comments from Reviewer 3** |  |
| The manuscript compared the content validity of two instruments used in measuring nurse pediatric pain knowledge and attitudes.  As strong points of the article, it should be noted that they have had a wide participation of experts. Furthermore, it appears that the author of the questionnaire, Manworren, has been involved in the counseling process.  As weaknesses, the authors acknowledge both in the limitations section and in the conclusions that, as future lines, a validation of the criterion and the construct is required.  From my point of view, we must differentiate between (Lobiondo-Wood G, Haber J.; 2013)^[1]^:   - The **content validity**. Content validity evaluates qualitatively whether the questionnaire covers all the dimensions of the phenomenon that wants to measure, since it is considered that an instrument is valid in its content if contemplates all related aspects with the concept that measures. The researcher begins by defining the concept and identifying the attributes or dimensions of the concept. The items that reflect the concept and its domain are developed. - The **apparent validity**. It is a subtype of content validity. It is a face validity, which is a rudimentary type of validity that basically verifies that the instrument fives the appearance of measuring the concept. It is an intuitive type of validity in which colleagues or subjects are asked to read the instrument and evaluate the content in terms of whether it appear to reflect the concept the researcher intends to measure. Or if the elements included in an instrument are relevant. - **Criterion validity**. It is the degree of correlation between an instrument and another measure of the variable under study that serves as criterion or reference. - **Construct validity**. It is understood as the degree to which an instrument measures the bipolar evaluative dimension for which it was designed.   Actually, in the present article, what they do is to measure **apparent validity**. It is important because the acceptance of a scale by several people gives consistency when using it. However, apparent and content validity is a relevant method especially when designing an instrument. It is not so important when the instrument has been previously validated and used in different areas.  On the other hand, and as the authors say, these tools have already been validated and used in various studies. Knowing that its content validity has already been reviewed as they describe and explain. In that case, why have they revised the apparent validity again? Why haven't they gone a step further? Why have the criteria not been applied and reviewed?  For these reasons, I think the article does not reach the level required for a journal like PLOS ONE, and should be rejected.  From my point of view, the approach and content is well developed, but due to the characteristics of the tools used, these require a criterion validity, a construct validity, or a cross-cultural validation.  The authors summarize the main research question and key findings. Even, the authors identify other literature on the topic and explain how the study relates to this previously published research.  However, I would like to make some specific suggestions in the next point. | Thanks for the feedback. As you already know, a valid instrument is a reliable one but a reliable instrument does not guarantee validity. Thus, validity is an important quality expected of instruments. As you rightly point out and explain, there are several types of validity (content, construct, criterion, discriminant among others). The choice on the type of validity to be assessed depends on the aim of this enterprise. Our reasons for assessing content validity as the starting point for other types of validity and reliability in the future have been provided in the manuscript (on page 2, lines 25-27; page 4, lines 92-95 and page 5, lines 96-99). It is significant to also mention that, the changes which have been made in the instruments would have been missed if we had not assessed their content validity as the starting point for other types of validity and reliability assessments to be explored in the future. |
| **Specific Comments from Reviewer 3** |  |
| Suggestions for improvement do not refer to major issues |  |
| Title |  |
| As a suggestion, and to contextualize more the article from the first moment, the title could refer to Pediatric Healthcare Providers' (HCPs) or pediatric nurses as well as keywords.  In fact, HCPs are referenced every time in the page 3, line 61 paragraph.  Another more clarifying explanation is found in page 3 and line 68: “These two instruments were developed to measure healthcare professionals and students’ knowledge and attitudes regarding children’s pain [13].” But it is still a personal appreciation. | The titles (long and short) and keywords have been amended; refer to page 1, lines 1-7 and page 3, line 54. |
| Abstract and introduction |  |
| Page 2; line 24. The introduction does not set the stage adequately. As in the title, it is required to specify who the study is aimed at. To miss this information may decontextualize and imply the study population.  In the abstract, the authors explain why the study matters and put the research in context properly. However:  In page 2; line 25; the authors clarify that “*This was considered necessary due to the universal differences in* ***culture, semantics and healthcare resources*** *in different parts of the globe*”, but in the following paragraph:  In page 2; line 31; they specify that the experts only will check **the relevance** and **clarity** of the items will be reviewed **without mention** culture, semantics and healthcare resources. If it is so important, because you mention it at the beginning of the abstract, could the experts have been asked about the changes due to the cultural factor? Has any change been made due to semantic and cultural changes? | Thanks for the feedback. We however disagree with this comment as the introduction provides the background information and justification for the current study. We appreciate your suggestion to include the target population as part of the title but this would make it too lengthy as the PNKAS was specifically designed for nurses whilst the PHPKASRP was directed at pediatric healthcare professionals in general.  Changes have been made to the assessable areas to reflect what happened in practice; this has been provided on page 6, lines 130-133.  At the end of the validation process, five items each were modified on the PNKAS and PHPKASRP as outlined on page 11, lines 249-251. Some of these changes were related to semantics (for instance, question 37 of PNKAS and questions 11 and 37 of PHPKASRP) and cultural changes (for instance, questions 7 and 36 of PNKAS and questions 10 and 36 of PHPKASRP). |
| Content validity is a relevant method especially when designing an instrument. It is not so important when the instrument has been previously validated and used in different areas. However, when an instrument is translated into another language, if the explored concepts are supposed to change significantly from one culture to another, it may be useful to recheck the face validity.  Moreover, in page 4, line 92 It is said that validity is not the property of an instrument, but depends on the interpretation related to the context and participants. However, knowledge of health is based on science, evidence, principles, theories, and is universal. Therefore, they do not depend on the interpretation of people and cultures.  Content validity evaluates qualitatively whether the questionnaire covers all the dimensions of the phenomenon to be measured, since an instrument is considered to be valid in its content if it considers all aspects related to the concept it measures. For this, it is necessary to have a clear idea of the conceptual aspects to be measured. And in this case, since the instrument had previously been validated and used in different areas, the content was already available. | Thanks for the feedback. As you already know, a valid instrument is a reliable one but a reliable instrument does not guarantee validity. Thus, validity is an important quality expected of instruments. As you rightly point out and explain, there are several types of validity (content, construct, criterion, discriminant among others). The choice on the type of validity to be assessed depends on the aim of this enterprise. Our reasons for assessing content validity as the starting point for other types of validity and reliability in the future have been provided in the manuscript (on page 2, lines 25-27; page 4, lines 92-95 and page 5, lines 96-99). It is significant to also mention that, the changes which have been made in the instruments would have been missed if we had not assessed their content validity as the starting point for other types of validity and reliability assessments to be explored in the future. |
| Page 3; line 67. This is what I cannot understand. In this section, the questions that arise are: When talking about the revised version (PHPKASRP). Is the article review being done for the first time in this article? Or has this version already been created before? Why do you call short version if they have almost the same number of questions?...May be could you give more details or change the way to explain it. | Changes have been made as found on page 4, line 71. |
| In the next sentence: page 3; line 68. “These **two instruments** were developed to measure healthcare professionals and students’ knowledge and attitudes regarding children’s pain (**13**)”. We find the reference of the PNKAS, what about the PHPKASRP reference? Could you explain this better? | The reference for PHPKASRP has been provided on page 4, lines 72-73. |
| When talking about instrument validation, I would give more information about whether or not PHPKASRP is validated, etc. | Information on the validity of PHPKASRP has been provided on page 4, lines 85-87. |
| Page 4; line 84. References for PHPKASRP are again needed. | Citation of this instrument has been provided on page 4, lines 87. |
| Figures and tables |  |
| The information in Tables 1 and 3 are explained in the text. Table 1 could be omitted by completing the information in the text. However, Table 3 can be kept, since it serves as a synthesis of the results. | As suggested, Table 1 has been omitted whilst Table 3 has been retained as the only table within the manuscript. Table 2 has been designated as S1 Table in the supplementary file named “S1_Table.docx”. |
| Methods |  |
| Page 6; paragraph line 128-133.There is no specific reference to cultural and semantic factors. Despite the emphasis that has been given in the introduction by providing references.  They talk about: comprehensiveness, objectivity, organization and relevance defined as “comprehensive: that issue containing important information to reach the objective of the study, stated in a comprehensible manner; Objective: that issue which is easy to understand; organization: the disposition of the issues and alternatives as well as their content; Relevant: that question which is related to achieving the goal of the research” | Changes have been made to reflect what occurred in practice, refer to page 6, lines 130-133. |
| Page 8; line 182. Again, the article reference of the revised version (PHPKASRP instrument) is missed. | Citation to this instrument has been provided on page 8, lines 182-183. |
| Results, discussion, conclusions |  |
| The results on page 10 should reference Table 3, where the results are summarized.  The authors acknowledge both the limitations and the conclusions that, as future lines, a validation of the criterion and the construct is required. | References to the now “Table 1” has been provided on page 10, lines 230-231. |
| Statistical analysis |  |
| This type of validity requires basic statistics. Experts and / or researchers use in evaluating the relevance of a scale that requires descriptive analysis. | Due to the confusion introduced by the mention of “by pediatric experts”, this phrase has been removed to enhance clarity. Refer to page 7, line 152. |

1. Lobiondo-Wood G, Haber J. Nursing research: methods and critical appraisal for evidence-based practice. 8th ed. St. Louis: Mosby; 2013.
