# Supplementary material for: Evaluating the content validity of two versions of an instrument used in measuring pediatric pain knowledge and attitudes in the Ghanaian context
Source: PLoS One. 2020 Nov 6;15(11):e0241983. doi: 10.1371/journal.pone.0241983 (PMC7647094; doi:10.1371/journal.pone.0241983)
Supplement: Attachment — Submitted filename: PONE-D-20-05637R1 Review.docx [file pone.0241983.s008.docx]

**REVIEWER COMMENTS ON MANUSCRIPT “Evaluating the content validity of two versions of an instrument used in measuring pediatric pain knowledge and attitudes in the Ghanaian context”**

**(PONE-D-20-05637R1)**

**Dear Editor**

Thank you again for the opportunity to review this manuscript. The manuscript aims to evaluate and compare the content validity of PNKAS and its revised version, PHPKASRP from Ghanaian perspective. The authors have done an excellent work to address the comments I raised in the first review. However, there are still some relatively minor comments that need to be addressed. Please find them below.

**Introduction**

Lines 70-73. “*Key among them are Pediatric Nurses’ Knowledge and Attitudes Survey regarding pain (PNKAS) [13] and 71 its revised version, the Pediatric Healthcare Providers’ Knowledge and Attitudes Survey Regarding Pain (PHPKASRP) [Manworren RCB, personal communication, August 16, 2018].*”

According to the authors, the developer is yet to publish the PHPKASRP. However, it has been almost two years since the developer provided the authors with the PHPKASRP. I’m wondering why the PHPKASRP was not published yet and the reasons behind that, although, according to the authors, “*it has been translated into other languages and permitted for use in many organizations around the world*”, and was described as a “*key*” instrument.

I think the PHPKASRP, until this date, does not reach the level to be a “key” instrument since there is no single publication about it, except for the abstract (ref. 17), or about its psychometric properties in English and other languages.

Lines 85-89. “Content validity of the PHPKASRP has been established by national content experts comprising of physicians, pediatric nurses and pharmacists in the United States of America [Manworren RCB, personal communication, August 16, 2018]. According to the developer, the instrument has been translated into other languages and permitted for use in many organizations around the world [Manworren RCB, personal communication, August 16, 2018].

Further, I do not think it would appropriate to state such information based on personal communication alone and not based on published evidence. The authors need to remove these statements or cite them properly.

**Discussion**

Lines 282-285. “*In line with the recommendations of Delgado-Rico and colleagues [43], decisions on items (i.e., elimination, modification or conservation) were made on the basis of the content validity indices, feedback given by experts, inputs from the instrument developer and the contribution of the items to the overall construct under investigation*”.

I still think you need to cite Delgado-Rico and colleagues in your methods since you followed their recommendations in your decisions making. The authors need to explain the reasons for not citing them if they disagree with that.

**Appendices**

There are several spelling mistakes in “S1 Appendix. Revised Pediatric Nurses’ Knowledge and Attitudes Survey regarding pain (r-PNKAS)”. For example, items 8, 10, 22, 23, and 33. Please correct them and check the rest of this document and other documents carefully.
