# Supplementary material for: Evaluating the content validity of two versions of an instrument used in measuring pediatric pain knowledge and attitudes in the Ghanaian context
Source: PLoS One. 2020 Nov 6;15(11):e0241983. doi: 10.1371/journal.pone.0241983 (PMC7647094; doi:10.1371/journal.pone.0241983)
Supplement: Attachment — Submitted filename: Response to Review Comments_01-10-2020.docx [file pone.0241983.s009.docx]

**Response to Review Comments**

Thank you for taking time off your busy schedules and providing us constructive feedback to further improve our manuscript. Please find below our responses to the review comments.

**Overall Comments from Reviewer 1**

Reviewer #1: I just have to congratulate the authors for their work on modifications. They have carefully read and responded to each and every reviewer review and comment. I am very satisfied with the final work, and among all I think that the first version has improved a lot.

**Response**

Thank you.

**Overall Comments from Reviewer 2**

Reviewer #2: I thank the authors for the improvements they have made to the manuscript. However, I still have some concerns related to my original comment in the first review.

**Response**

Thanks for accepting to review our manuscript. Responses to the additional concerns have been provided below.

**Overall Comments from Reviewer 3**

Reviewer #3: Dear Editor
Thank you again for the opportunity to review this manuscript. The manuscript aims to evaluate and compare the content validity of PNKAS and its revised version, PHPKASRP from Ghanaian perspective. The authors have done an excellent work to address the comments I raised in the first review. However, there are still some relatively minor comments that need to be addressed. Please find them below.

**Response**

Thank you all for the feedback. Please find below our responses to the concerns raised.

**Specific Comments from Reviewers 1-3**

**Comment**

**Introduction**

Lines 70-73. “Key among them are Pediatric Nurses’ Knowledge and Attitudes Survey regarding pain (PNKAS) [13] and 71 its revised version, the Pediatric Healthcare Providers’ Knowledge and Attitudes Survey Regarding Pain (PHPKASRP) [Manworren RCB, personal communication, August 16, 2018].”

According to the authors, the developer is yet to publish the PHPKASRP. However, it has been almost two years since the developer provided the authors with the PHPKASRP. I’m wondering why the PHPKASRP was not published yet and the reasons behind that, although, according to the authors, “it has been translated into other languages and permitted for use in many organizations around the world”, and was described as a “key” instrument.
I think the PHPKASRP, until this date, does not reach the level to be a “key” instrument since there is no single publication about it, except for the abstract (ref. 17), or about its psychometric properties in English and other languages.

**Response**

Thanks for the feedback. We do not know why the instrument developer (Manworren, R.C.B) has not yet published findings from the PHPKASRP. She however, indicated in one of our email correspondences that our work has served as a reminder for her to publish findings related to the PHPKASRP. We have amended the statement relating to the PHPKASRP as a “key” instrument on the subject. Refer to page 3, lines 66-71.

**Comment**

Lines 85-89. “Content validity of the PHPKASRP has been established by national content experts comprising of physicians, pediatric nurses and pharmacists in the United States of America [Manworren RCB, personal communication, August 16, 2018]. According to the developer, the instrument has been translated into other languages and permitted for use in many organizations around the world [Manworren RCB, personal communication, August 16, 2018].
Further, I do not think it would appropriate to state such information based on personal communication alone and not based on published evidence. The authors need to remove these statements or cite them properly.

**Response**

Amendments have been made to reflect the state of affairs regarding these issues. Refer to page 4, lines 85-88.

**Comment**

**Discussion**

Lines 282-285. “In line with the recommendations of Delgado-Rico and colleagues [43], decisions on items (i.e., elimination, modification or conservation) were made on the basis of the content validity indices, feedback given by experts, inputs from the instrument developer and the contribution of the items to the overall construct under investigation”.
I still think you need to cite Delgado-Rico and colleagues in your methods since you followed their recommendations in your decisions making. The authors need to explain the reasons for not citing them if they disagree with that.

**Response**

As suggested, Delgado-Rico and colleagues have been cited under the materials and methods section on page 8, lines 173-176.

**Comment**

**Appendices**

There are several spelling mistakes in “S1 Appendix. Revised Pediatric Nurses’ Knowledge and Attitudes Survey regarding pain (r-PNKAS)”. For example, items 8, 10, 22, 23, and 33. Please correct them and check the rest of this document and other documents carefully.

**Response**

Spelling errors have been corrected in the “S1_Appendix” and “S2_Appendix” in the revised documents.

**Overall Comments from Reviewer 4**

Reviewer #4: The authors did a great job in adjusting the manuscript in response to the reviewers' comments, but a few issues remain. A main issue that remains for me is that the current data presented on its own isn't very strong and I still believe the current manuscript would be stronger if the authors could add data on further validation studies with the modified instruments.

**Response**

Thanks for the feedback. We humbly disagree with the comments relating to the strength of the current manuscript due to the absence of other validation studies as there is no published recommendation on the number of validation studies to be included in a manuscript to qualify for the supposed strength. This notwithstanding, we acknowledge the importance of additional psychometric testing (validity and reliability) of the modified instruments which have been stated in the discussion (on page 33, lines 345-347) and conclusion (on page 33, lines 356-357) sections of our manuscript. As indicated in the recommendations, other types of validity and reliability of the modified instrument would be pursued in the future.

**Specific Comments from Reviewers**

**Comment**

In the introduction, p. 4 line 84, it is unclear to me that the PHPKASRP is a variation of the previously discussed instrument. That only becomes clear to me on p. 8 line 180. Therefore, I would suggest including this information earlier on.

**Response**

Information on the above have been provided earlier on at page 3, lines 67-71.

**Comment**

The data analyses section does not provide any details on what is being done with the comments made by the participants on appropriateness to the culture, semantics (comprehensibility, simplicity, grammatical construction) and healthcare resources available in the Ghanaian context. It only becomes clear in the results that the purpose of these comments is to redesign these instruments. More detail on how these comments were systematically dealt with to redesign the instruments is needed.

**Response**

Details on the application of the comments in redesigning the instruments have been provided on page 8, lines 183-189.

**Comment**

I find it strange that the development of the PNKAS and PHPKASRP instruments is detailed at the end of the methods sections, I would prefer to see this more upfront, might even fit in the introduction.

**Response**

As suggested, information on the instrument development processes has been moved upfront. Refer to page 5, lines 99-120 and page 6, lines 121-132.

**Overall Comments from Reviewer 5**

**Comment**

I highly recommend you to discuss and take in account previous articles that purpose questionnaires for evaluation of Back Pain. In a quick search I found some articles that can be improve your introduction as well as the discussion, as follow:

Spanish translation, cross-cultural adaptation and validation of the Argentine version of the Back Pain Attitudes Questionnaire Pierobon, A., Policastro, P.O., Soliño, S., (...), Raguzzi, I.A., Villalba, F.J. 2020 Musculoskeletal Science and Practice
46,102125

Is There Equivalence between the Electronic and Paper Version of the Questionnaires for Assessment of Patients with Chronic Low Back Pain? Azevedo, B.R., Oliveira, C.B., Araujo, G.M.D., (...), Pinto, R.Z., Christofaro, D.G.D. 2020 Spine
45(6), pp. E329-E335

Back Pain and Body Posture Evaluation Instrument (BackPEI): Development, content validation and reproducibility Noll, M., Tarragô Candotti, C., Vieira, A., Fagundes Loss, J. 2013 International Journal of Public Health
58(4), pp. 565-572

Psychometric Study and Content Validity of a Questionnaire to Assess Back-Health-Related Postural Habits in Daily Activities Monfort-Pañego, M., Miñana-Signes, V. 2020 Measurement in Physical Education and Exercise Science

Validation of the Japanese Version of the Fremantle Back Awareness Questionnaire in Patients with Low Back Pain Nishigami, T., Mibu, A., Tanaka, K., (...), Stanton, T.R., Moseley, G.L. 2018 Pain Practice
18(2), pp. 170-179

**Response**

Upon reading the suggested articles, some clarifications have been made in the discussion section. Refer to page 31, lines 297-299, 311-313.
